# How should health systems prepare for the evolving COVID-19 pandemic? Reflections from the perspective of a Tertiary Cancer Center

**DOI:** 10.6061/clinics/2020/e1864

**Published:** 2020-04-06

**Authors:** Andre Tsin Chih Chen, Camila Motta Venchiarutti Moniz, Ulysses Ribeiro-Júnior, Maria Del Pilar Estevez Diz, João Victor Salvajoli, Karina Gondim Moutinho Da Conceição Vasconcelos, José Otávio Costa Auler-Júnior, Ivan Cecconello, Edson Abdala, Paulo Marcelo Gehm Hoff

**Affiliations:** I Instituto do Cancer do Estado de Sao Paulo (ICESP), Hospital das Clinicas HCFMUSP, Faculdade de Medicina, Universidade de Sao Paulo, Sao Paulo, SP, BR; II Divisao de Anestesiologia, Hospital das Clinicas HCFMUSP, Faculdade de Medicina, Universidade de Sao Paulo, SP, BR; III Disciplinas de Cirurgia do Aparelho Digestivo e Coloproctologia, Hospital das Clinicas HCFMUSP, Faculdade Medicina, Universidade de Sao Paulo, Sao Paulo, SP, BRChen ATC, Moniz CMV, Ribeiro-Júnior U, Diz MPE, Salvajoli JV, Vasconcelos KGMC, et al. How should health systems prepare for the evolving COVID-19 pandemic? Reflections from the perspective of a Tertiary Cancer Center. Clinics. 2020;75:e1864

In early December 2019, a pneumonia of unknown origin was first diagnosed in Wuhan, China 1. The pathogen was identified as a novel coronavirus and was subsequently named severe acute respiratory syndrome coronavirus 2 (SARS-CoV-2) 2. On January 30^th^, 2020, The World Health Organization (WHO) declared coronavirus disease 2019 (COVID-19) a public health emergency of international concern 3. As of March 18^th^, 2020, there were 212,616 laboratory-confirmed cases worldwide 4. Governments have responded in various ways to this evolving public health emergency 3,5,6.

In light of the emerging data 1-3,6,7, we discuss issues to be considered as health systems systematize their responses. We are not trying to predict the future, but rather, present potential scenarios. Herein, we provide the perspective of the Instituto do Câncer do Estado de São Paulo (ICESP) - Hospital das Clínicas da Faculdade de Medicina da Universidade de São Paulo, a publically funded tertiary referral cancer center in Brazil. The current editorial does not represent institutional or governmental positions. Formal policies take time to receive official approval. Because of the urgent need for local, regional, and national preparation, we write collectively as free thinkers with the best interests of society in mind, and as responsible doctors that have vowed to provide the best care for our patients 8.

The Spanish Influenza pandemic of 1918-1919 has provided us with uncontrolled populational data on the importance of social measures for mitigating the harm of epidemics. Historical work 9 and mathematical modelling studies 10 suggest that early and sustained implementation of such measures significantly reduced mortality rates during the 1918-1919 epidemic in the United States.

The SARS-CoV-1 2002-2003 epidemic has also taught us valuable lessons. As stated in the WHO consensus report, “the experience in affected areas has shown that the transmission can be prevented by adherence to basic public health measures, including rapid case detection, case isolation, contact tracing and good infection control, including handwashing and the use of personal protective equipment” 11. Prevention and case isolation are perhaps the most critical steps to prevent any given epidemic, and require active effort from authorities at all levels. These non-pharmacologic interventions play an important role in mitigating a community-wide epidemic, by 1) delaying the exponential growth of the curve, 2) reducing the number of cases, and 3) spreading the total demand over a longer period of time to match the capacity of hospitals and other healthcare settings 10.

Social institutions like churches have played an essential role in shifting the general public mindset from debauchery or indifference toward prevention. Practical measures have been implemented, including the prohibition of kisses and holding hands during mass 12,13 and avoiding common vessels during eucharistic communion 14. Social greetings like kisses and handshaking should be avoided in any environment, especially in hospitals.

In a recent publication, the China Medical Treatment Expert Group for COVID-19 reported on the epidemiology and clinical outcomes of 1099 laboratory-confirmed COVID-19 cases 1. Severe illness occurred in 15.7% of the patients after admission to the hospital, 5.0% were admitted to the ICU, 2.3% underwent invasive mechanical ventilation, and 1.4% died. Severe disease was associated with comorbid disorders and older age. Of note, in patients with comorbidities, early estimates of the case-fatality rate were up to 73.3%, Data Supplement, Table S3 from Guan et al. 1. Only 0.9% of the cohort had cancer.

At this moment, it seems too early to tell if these estimates are generalizable or even accurate, but caution is warranted for patients with comorbid disorders. If real mortality parameters are within the range of published data, there is a significant risk of severe events, and every effort should be taken to guarantee that suspected cases receive an adequate evaluation. Complexity is added to the triage by the fact that only 43.8% had fever on hospital admission 1 and by extensive differential diagnoses in cancer patients such as febrile neutropenia, antitumor drug side effects, and other infections.

Regarding cancer patients, follow-up consultations and exams from patients with no active disease or treatment could be postponed to reduce the risk of exposure. Patient companions should consider going to the hospital only when necessary. Attention should also be given to food-courts and cafeterias, both within and nearby the hospital, given that healthcare workers and patients may share the same zone without proper protective equipment.

Before initiating systemic anticancer treatment, physicians should weigh the evolving COVID-19 epidemic risk. Factors like disease severity, the potential benefit from treatment, drug schema immunosuppression potential, patient age, and comorbid conditions should be considered. In the adjuvant or neoadjuvant scenarios, physicians should consider the pros and cons of every available treatment option. We should also consider prophylactic interventions as soon as consistent data demonstrate antiviral effects against SARS-CoV-2 in clinical trials, for either the patients, their households, or health care personnel.

Surgery is central in cancer treatment. Should there be an increase in the number of COVID-19 cases in Brazil, elective surgeries could be postponed. There are a limited number of intensive care units (ICUs), with an imbalance between the public and private sectors 15. Along with Brazilian authorities' efforts to improve the number of ICU beds during the COVID-19 outbreak, logistics need to be implemented to avoid contact between exposed and non-exposed patients. Non-life-threatening surgeries could be postponed until the epidemic is under control, both to avoid the risk of intercurrent COVID-19 infection and to guarantee ICU beds to critical patients. In cases with neoadjuvant treatment, the interval to surgery needs to be entered into the equation. It is estimated that 10% of patients affected by COVID-19 need prolonged admission in ICUs. This emphasizes the importance of non-pharmacologic community strategies to reduce incidence and lessen the need for healthcare services 10.

In any given epidemic, real-time quality data is essential for critical decision-making. The Brazilian Health Ministry is to be commended for implementing a REDCap online form for notification of suspected cases 16. Additional efforts are needed in systems integration, so that relevant information is available for healthcare workers and other stakeholders. We suggest that the Brazilian Health Ministry consider WHO recommendations 17 and lead the implementation of standard online forms to collect sensitive data on clinical characteristics, treatment, and outcomes.

Of note, Brazil's legislative bodies are to be commended for the fast approval of laws to fight the COVID-19 international public health emergency. The Federal Council of Medicine (Conselho Federal de Medicina) and Ethics Committees are to interpret the new law and clarify if consent forms to diagnose and treat COVID-19 patients can be waived 18. Should consent be needed, we suggest verbal consent as a means to prevent pen and paper consent forms from becoming a transmission route, as we have seen in the Diamond Princess situation 19.

At this point, it is appropriate to comment on the fear that excessive data may lead to panic among the general public. We partially agree with this statement, though we also stress that sensitive data need to be readily available to healthcare providers and decision-makers in order to mitigate this epidemic. Misinformation erodes public trust and may result in backlash 20.

As the number of new cases accrue in Brazil, physicians and healthcare workers on the front-line should have their non-clinical workload reduced to a minimum, to focus on adequate contact precautions and patient care. Healthcare providers should reallocate administrative personnel to perform non-clinical tasks including paperwork-filling and data collection as much as possible. Hospitals should take their time to train staff to effectively implement contact precautions and flow processes. Adequate emotional support for staff and reasonable hours of risk exposure are also suggested to prevent burnout, as health professionals struggle to care for patients and protect their lives and families.

We hope this editorial may foster discussion among the authorities and healthcare stakeholders, and help to plan and implement pathways to minimize the impact of the COVID-19 epidemic.

## AUTHOR CONTRIBUTIONS

Chen ATC was responsible for the study main idea and conception, data acquisition, analysis and interpretation, manuscript drafting and final approval of the version to be published. Moniz CMV was responsible for the data acquisition and interpretation, manuscript critically review for intellectual content and final approval of the version to be published. Ribeiro-Júnior R, Diz MPE, Salvajoli JV, Vasconcelos KGMC, Auler-Júnior JOC, Abdala E and Cecconello I were responsible for the data interpretation and manuscript critically review for intellectual content and final approval of the version to be published. Hoff PMG was responsible for mentoring, data interpretation, manuscript critically review for intellectual content and final approval of the version to be published.
